# The ART of evidence-based care: proof of concept of a novel strategy to accelerate research translation in health organisations

**DOI:** 10.3389/frhs.2025.1691307

**Published:** 2026-01-12

**Authors:** Katherine E. Harding, Annie K. Lewis, Julie Considine, Penny Casey, Ian D. Davis, Amy Dennett, Germaine Tan, Jacqueline A. Boyle, Nicholas F. Taylor

**Affiliations:** 1 La Trobe University, Melbourne, VIC, Australia; 2 Eastern Health, Box Hill, VIC, Australia; 3 Deakin University, Melbourne, VIC, Australia; 4 Monash University, Melbourne, VIC, Australia

**Keywords:** implementation science, knowledge translation, evidence-based practice, quality improvement, health services, professional education

## Abstract

**Introduction:**

This study evaluated a novel strategy to improve accessibility of implementation science to clinicians in a large health network. The strategy, called Accelerating Research Translation (ART), was based on an adaptation of the A3 problem-solving method developed for use in healthcare quality improvement.

**Method:**

An observational pre-post design was used to conduct a ‘proof-of-concept’ trial of the ART strategy. Clinicians who identified a gap between a clinical practice guideline and usual care were recruited as Project Leads to conduct research translation projects over a 6-month period, supported by a training programme involving a workshop series and mentor. The proportion of patients receiving guideline-consistent care for each project was measured pre- and post-intervention, and results across projects were combined in a planned pooled meta-analysis. Secondary outcomes included changes in confidence in research translation for Project Leads and the degree of evidence of implementation, mapped against Meyer and Goes' stages of healthcare implementation.

**Results:**

Ten projects were completed by 13 Project Leads, involving a diverse range of professional disciplines, clinical specialties, and healthcare settings. Pooled analysis of data from 768 patients suggests the ART programme increased the odds of receiving evidence-based care (logOR 2.52, 95% CI: 0.93–4.11, *p* < 0.01). Effects were stronger for evidence–practice gaps that could be addressed with changes to local policies and processes than those dependent on changing behaviour or complex systems. Confidence of Project Leads improved, and all reported increases in the level of implementation of evidence.

**Conclusion:**

This novel approach empowered clinicians to tackle evidence–practice gaps within a familiar structure already well-accepted and understood by health service providers. This concept provides a promising mechanism to demystify implementation science and address local evidence–practice gaps at scale.

## 1 Introduction

Generating and publishing evidence is not sufficient to change practice in healthcare. Addressing confidence and knowledge gaps among health professionals in the science of implementing evidence into practice is important for accelerating the uptake of evidence (1–3), but this will only be successful if support structures and tools are in place within health services to encourage and facilitate this work. While enquiry work falling into the domains of ‘research' or ‘continuous improvement’ is typically well recognised as part of the healthcare landscape, ‘evidence translation’ is not always acknowledged or embraced within the established support and governance structures in health services (4).

One potential way forward is to deliberately integrate education and support for research translation into existing quality improvement structures within large health organisations. The expectation that clinicians integrate service improvement work into their usual practice is often already well-established within healthcare roles. Improvement activities are sometimes conducted within the context of formal training programmes (5) or may be included as a standard expectation within clinician roles (6, 7). Redirecting some of the time typically allocated to traditional improvement work specifically into projects that target gaps between evidence and clinical practice has the potential to improve patient safety and quality of care with minimal need for additional resources. We propose that four elements required to facilitate targeted research translation work in health services are already in place but need to be brought together in a cohesive, formalised way to realise this potential.

Firstly, high-quality clinical practice guidelines provide a ready source of recommendations based on the latest evidence and synthesised by a panel of international experts for a wide range of healthcare interventions (8). Such guidelines aim to promote health, prevent harm, encourage best practice, and reduce waste and are therefore well-aligned with the core business of healthcare organisations and their quality improvement endeavours. The increasing accessibility of high-quality evidence summaries with clear recommendations for practice is an important first step for the translation of research into practice.

Secondly, there is an abundance of information to guide individuals and organisations on the best methods to implement research into practice (9). Various terms have been used interchangeably to describe this process, including research translation, knowledge translation, and evidence implementation. For the purpose of this study, we use the term research translation, which sits within the broader field of implementation science describing the scientific study of methods and strategies to facilitate research translation. More than a decade ago, Tabak et al. (10) described 61 different implementation models for enhancing the dissemination and implementation of research evidence. However, for these tools to be effective, clinicians need guidance on how, when, and why to use them. Attempts to synthesise, evaluate, and categorise these tools have made an important contribution to the field of implementation science (9, 11), but the complexity of information creates challenges in making research translation theory and strategy accessible for health professionals to apply within the context of daily practice. Implementation scientists have an important role in continuing to push the boundaries and drive new learning in this field, but to bring about change on the scale required, we need simple ways to put this research translation knowledge and theory in the hands of those delivering health services.

Thirdly, healthcare organisations already have structures to guide enquiry and reporting that can be used as a scaffold for deliberate research translation initiatives. In most organisations, these currently take labels of either ‘research’ or ‘continuous improvement’, each with its own support structures, reporting processes, and governance requirements. Most healthcare organisations have existing reporting lines for quality improvement projects, and ethical review committees provide an additional level of oversight for work involving the collection and reporting of human data that is beyond what could be reasonably expected within the course of usual clinical care (4). Research translation initiatives do not necessarily have a natural home with either one of these paradigms but have much in common with both; skills and tools often associated with performance improvement can be of value in identifying barriers and changing behaviour, whereas a scientific approach to the appraisal of evidence and outcome measurement is also vital when implementing and evaluating research translation initiatives. While existing structures may require adaptations, in many health organisations, there will be opportunities to build on these foundations to promote targeted work to facilitate the implementation of evidence into practice.

Finally, health service organisations commonly promote the use of a range of tools and standards to support quality improvement work that have potential for adaptation to research translation projects. Tailoring these familiar tools for research translation projects provides a novel opportunity to accelerate the translation of research into practice.

This proof-of-concept project aimed to test a strategy developed by the authors and titled ‘Accelerating Research Translation’, otherwise known as the ART strategy, that builds on these opportunities to support clinicians to undertake research translation projects within a large health organisation. The primary aim was to determine whether implementation of ART would be associated with an increase in the proportion of patients receiving care in accordance with national or international evidence-based clinical practice guidelines in the selected clinical areas. Secondary aims were to provide insights into confidence in research translation for those who participated in the initiative and the degree to which the evidence was implemented at the project sites.

## 2 Methods

### 2.1 Study design

An observational pre-post design was used to conduct a ‘proof-of-concept’ trial of the ART strategy. Clinicians who identified a gap between a recommendation in a clinical practice guideline and current practice in their workplace were recruited to conduct approximately 10 research translation projects using the ART strategy. Each project was supported by online resources, group training, and a mentor. The proportion of patients receiving care in accordance with the chosen guideline recommendation before and approximately 6 months after implementing their project was measured for each project, and findings were combined in a planned pooled analysis across projects. The study was approved by the Eastern Health Human Research Ethics Committee (approval number LR23-017-94719).

### 2.2 Study setting

The study was conducted at Eastern Health, a large metropolitan healthcare network in Melbourne, Australia, between July 2023 and July 2024. The network incorporates three large tertiary care hospitals, two subacute care inpatient facilities, two small hospitals offering limited inpatient services, and a range of sites providing ambulatory care. The work was supported by a philanthropic research grant from the Eastern Health Foundation.

### 2.3 Participants

The leads of each of the implementation projects (‘Project Leads’) were defined as participants in the project and provided written informed consent. Clinicians were eligible to participate as Project Leads if they were (a) employed in the health organisation; (b) endorsed by their manager to attend the training and undertake their proposed project; (c) able to identify a gap between routine care in their workplace and a recommendation in a nationally or internationally recognised clinical practice guideline; and (d) motivated to participate in the training programme, lead their proposed project, and collect the required data. After completing an application form, Project Leads were purposively selected to include a range of disciplines from allied health, nursing and midwifery, and medicine with the aim of achieving gender diversity both among Project Leads and the patient groups impacted by the proposed implementation projects. Clinicians had the option to co-lead projects with a colleague if they wished.

### 2.4 Intervention

The ART strategy was designed as a structured, evidence-based approach to support clinicians in addressing evidence–practice gaps in their workplace. It aimed to guide participants through the steps of appraising a clinical practice guideline (including its applicability to the local context), understanding barriers and facilitators to implementation, identifying and executing evidence-based implementation strategies, and evaluating the outcomes.

Central to the intervention was the ART-A3, an adaptation of the widely used A3 problem-solving tool. The A3 approach to problem-solving was originally developed by Toyota as part of its lean methodology and takes its name from the aim of visualising the process by documenting, analysing, and proposing solutions to problems on a single sheet of A3 paper. The approach has been widely adopted in health services to define and analyse problems, identify root causes, design interventions, and plan evaluations (12, 13). The version of the tool previously used in this health service for quality improvement projects comprised eight steps: (1) identify a problem/idea; (2) set a target or goal; (3) understand the current situation; (4) identify and analyse root causes; (5) develop the target or future state; (6) implement solutions (typically using Plan–Do–Study–Act cycles); (7) evaluate the change; and (8) standardise and share success.

The intention in creating the ART-A3 was to preserve the familiarity of the existing health service quality improvement A3 template while adapting key items to guide staff through the processes that need to be considered when implementing evidence into practice. Tailoring of the ART-A3 as a tool for research translation included a focus on defining the evidence practice gap in step 1 (including analysis of the strength and applicability of the evidence), an emphasis on barriers and facilitators influencing implementation of the evidence into practice in steps 3 and 4, and the use of implementation science frameworks to guide actions in steps 5 and 6. A more detailed description of the ART-A3 can be found in Supplementary Figure S1.

Participants were supported by four 2.5 h workshops, covering topics such as evidence appraisal, introduction to the ART-A3 tool, project planning, implementation science theory, and project evaluation and structure to align with the eight steps of the ART-A3 template (Supplementary Table S1). Sessions 1–3 were conducted over 2 months at the beginning of the study period, with participants expected to carry out activities between sessions to progress their project. Session 4 was conducted 6 months later, to guide participants through the process of analysing their data and to plan the dissemination of findings. The sessions were delivered by an experienced health service researcher with postgraduate qualifications in implementation science, using a mix of in-person and online formats. Recordings of each session were made available to participants who were unable to attend. Each Project Lead was allocated a mentor with experience in implementation science, and course materials and written resources were made available to participants through the health service's online platform for professional education.

Within the course content, participants were introduced to a range of implementation tools and frameworks. For example, the Consolidated Framework for Implementation Research (CFIR) (14) and the COM-B model of behaviour change (considering opportunity and motivation) (15) were introduced to guide investigation of the current state and prompt identification of barriers and facilitators. Michie et al.'s (15) behaviour change wheel and the Action, Actor, Context, Target, Time (AACTT) model were used to help participants select implementation strategies (16). These models were presented as tools that could act as prompts for identifying barriers and strategies to tackle them but were not intended to be prescriptive.

A variety of strategies were used to deliver the course content. Each workshop included some material explained using didactic teaching supported by slides, interspersed with interactive activities. For example, in session 2, an overview of the COM-B was provided in lecture format, and then participants were asked to divide into small groups, choose one project represented within their group, and brainstorm potential barriers to implementation of the clinical practice guideline recommendation of interest under the domains of capacity, opportunity, and motivation. Other activities involved all participants applying their learning to a hypothetical case study or reporting back on progress with their own project for group feedback and discussion. Slides, recordings of lectures, and supplementary reading materials were provided through an online platform. A more detailed description of the intervention is provided using the Template for Intervention Description and Replication (TIDieR) checklist in Supplementary Table S2 (17).

The Project Leads did not receive any financial assistance to participate in the programme or implement changes. Managers endorsed participation and provided time release for workshop attendance, and activities associated with implementing the projects were absorbed within the usual workload, consistent with other clinician-led healthcare improvement work at this organisation.

### 2.5 Outcome measures

The primary outcome was the change in the proportion of patients receiving care according to the selected clinical practice guideline recommendation after the implementation of each research translation project, compared with those receiving care pre-implementation. For each individual research translation project, one binary outcome was identified that indicated alignment of care with the clinical guideline. Data were most commonly obtained through an audit of routinely collected healthcare data pre- and post-intervention and collected retrospectively by each Project Lead from a sample of patients from a period spanning between 6 and 12 months prior to the first workshop. Although this process created potential for bias from lack of blinding in data collection, funding was not available for blinded assessors, and Project Leads were aided by local knowledge of each project setting. The risk was mitigated by the use of objective outcomes, such as counts of the patients who had specific interventions documented in their medical histories, and was guided by experienced mentors.

Secondary outcomes were collected at both the participant and project levels. Participant outcomes included changes in the confidence of participants in skills and knowledge related to research translation, self-reported by each Project Lead during workshops 1 and again at project completion using a questionnaire based on a tool designed by Young (Supplementary Table S3) (2). At the first workshop and project completion, the Project Leads also evaluated the state of implementation of evidence in their field against the stages of healthcare implementation (SHI) described by Meyer et al. (18). The wording for each intervention stage was adapted to reflect the intended purpose of implementation of evidence into practice (Supplementary Figure S2). One evaluation against the SHI was completed for each project, with ratings agreed by consensus for projects with joint Project Leads.

### 2.6 Analysis

Within each project, the primary outcome (representing the proportion of patients receiving care consistent with the relevant guideline recommendation) was analysed using odds ratios, log transformed for symmetrical distribution around zero. In cases where no patients were receiving evidence-based care, a 1 was imputed in place of 0 to enable computation of an odds ratio. Data for each of the projects were then combined using a prospectively planned pooled analysis (19). We planned to pool results in a planned meta-analysis because of relative clinical homogeneity (common intervention, common outcome of the proportion meeting clinical practice guidelines, and common population of patients of the same health organisation). All were asked to define a binary outcome that could be used to indicate whether a patient was receiving care aligned with the chosen guideline recommendation and measure this outcome pre- and post-intervention. These data were combined in a meta-analysis with a random-effects model with the result expressed following recommendations from the Cochrane Collaboration (20). Heterogeneity was analysed by observing the *I*^2^ value, with sensitivity analyses or subgroup analyses planned to explore sources of heterogeneity if *I*^2^ exceeded 50%. The target number of projects was based on resources and the availability of mentors for each project and was a suitable number for concurrent small group training. Within each research translation project, the target sample size was determined by the Project Leads based on calculations of the sample size required detect a clinically significant change in the proportion of patients receiving evidence-based care, with an alpha value of 0.05 and power at 0.8. For planned pooled analyses, it has been estimated that five or more studies (or in this case projects) are needed to consistently achieve power from random-effects meta-analyses that are greater than the studies that contribute to them, providing confidence that 10 projects would provide sufficient data for this analysis (21).

Secondary outcomes (research translation confidence and stages of healthcare implementation) were analysed descriptively, supplemented with exploratory comparisons of paired pre- and post-responses using the Wilcoxon signed-rank sum test. The study was underpowered for these outcomes but was sufficient to provide an estimation of effect size consistent with the purposes of this proof-of-concept study.

## 3 Results

### 3.1 Participants characteristics

Fourteen Project Leads were recruited to participate in the study, leading 11 projects: 8 with a single Project Lead and 3 projects co-led by two clinicians. Most Project Leads were female (*n* = 12, 86%) and represented a broad range of professional disciplines, clinical areas, and levels of professional experience (Table 1).

**Table 1:** Characteristics of Project Leads.

Participant characteristics	*n* (%)
Gender	
Female	12 (86)
Male	2 (14)
Professional group	
Registered nurse	2 (14)
Doctor	2 (14)
Physiotherapist	3 (21)
Occupational therapist	2 (14)
Dietitian	2 (14)
Medical imaging technologist	1 (7)
Podiatrist	1 (7)
Clinical support services	1 (7)
Clinical specialty	
Aged care	6 (43)
Cancer	1 (7)
Foot care	1 (7)
Intensive care	1 (7)
Nuclear medicine	1 (7)
Obesity	1 (7)
Obstetrics	1 (7)
Stroke	2 (14)
Level of experience	
<2 years	1 (7)
2–10 years	6 (42)
>10 years	7 (54)

**Figure 1:**
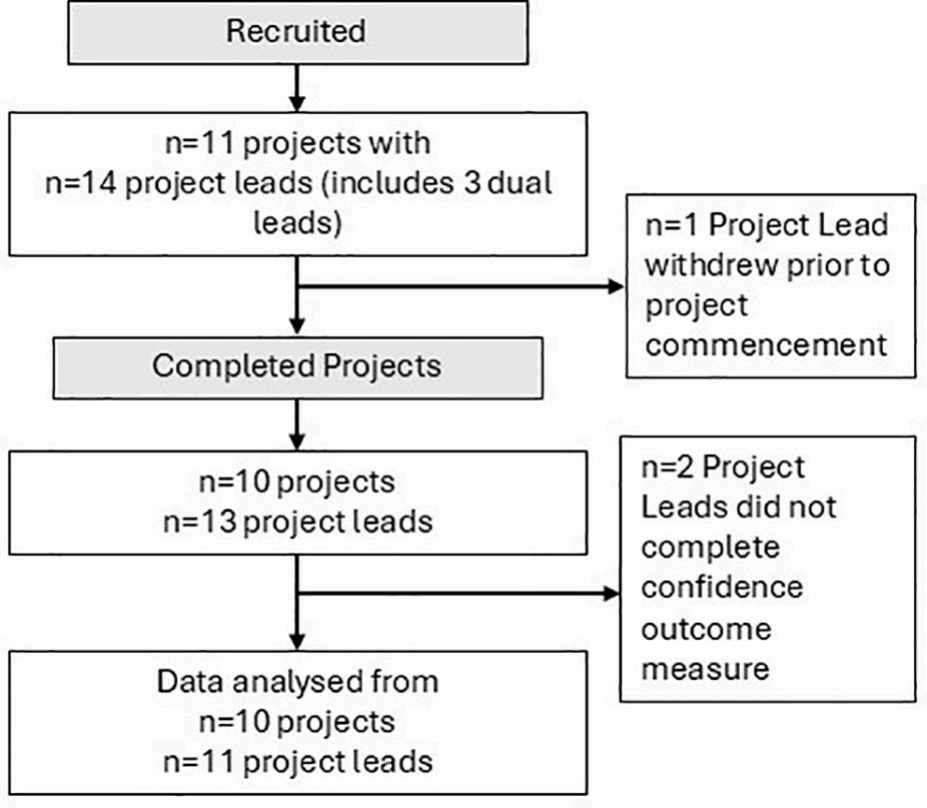
Flowchart of participants and projects through the trial.

Each of the workshops had an attendance rate of at least 85%, and no Project Lead attended fewer than three of the four workshops. One Project Lead withdrew after attending the workshops due to workload pressures but before the implementation of any evidence-translation strategies. Two others were on extended leave at the time of the final workshop and did not provide follow-up data on confidence in research translation, but both had co-Project Leads, and the projects were completed as planned (Figure 1).

### 3.2 Project characteristics

The evidence implementation projects were conducted in a range of clinical areas, focused on international and national guidelines in stroke, falls, nutrition, and surgical management (Table 2). Pooled baseline data collected by Project Leads prior to commencement of the projects identified that fewer than 25% of patients were receiving care in accordance with the specified guideline. One project was abandoned before implementation of any evidence-translation strategies, leaving 10 projects for inclusion in the analysis. Most projects were conducted in clinical areas with similar proportions of male and female patients, apart from one relating to neonatal care that aimed to improve care for women and their babies.

**Table 2:** Characteristics of completed projects.

Project number and setting	Evidence for implementation	Evidence source	Pre-implementation state	Implementation strategies used	Outcome measure
Psychogeriatric residential aged care	Use of visual menus to support meal choices for people living in residential care	Dietitians Australia Aged Care Quality Standards Toolkit (22)	No residents being offered this service to support meal choice	Resource development; staff training	% of residents offered a visual menu
				Integration of a visual menu into procedures for kitchen staff; demonstration; reminders	
2. Hospital nuclear medicine department	Time from sestamibi injection to parathyroid scintigraphy recommended 90–150 min	European Association of Nuclear Medicine practice guidelines (23)	Delayed phase imaging regularly occurred 3–4 h post-injection, times not always documented	Change to health service policy; staff training; visual resources; scheduling changes; correct procedures reinforced	% of scans within the 90–150 min interval
3. Bariatric dietetics clinic	Dietetics follow-up within 8 weeks after bariatric surgery	ANZMOSS Public Bariatric Surgery National Framework (24)	One-third of patients receiving review within recommended time, hindered by lack of clinic capacity	Protected capacity for first post op reviews, short-term backlog reduction activities, longer time between review appointments	% post op reviews within <8 weeks
4. Acute inpatient and rehabilitation settings	Cognitive assessment for all patients admitted with stroke prior to discharge	Australian Stroke Foundation guidelines (25)	<20% of patients receiving cognitive assessment; hindered by lack of confidence, resources, and time	Resource packages developed; staff education; promotion during clinical supervision; management support	% patients with assessment documented
5. Inpatient rehabilitation	Group circuit training for patients receiving inpatient rehabilitation after stroke	Australian Stroke Foundation guidelines (25)	Group circuit classes not offered in inpatient rehabilitation in this health service	Staff training; scheduling changes; recruitment of champions; integration into physiotherapy student programme	% patients who received circuit training
6. Acute medicine inpatient hospital ward	Targeted falls prevention education for patients in acute medical wards	World guidelines for falls prevention and management (26)	25% of patients had targeted falls prevention documented during acute admission	Staff education; recruitment of champions; visual prompts; reminders at team huddles; audit and feedback	% patients with education documented
7. Community rehabilitation	Gait speed and balance assessment for all patients presenting with falls	World guidelines for falls prevention and management (26)	<15% of patients met guidelines; impeded by therapist knowledge, environment, low priority	Staff training; use of champions; resources and equipment made available; new processes established; audit and feedback	% patients correctly assessed
8. Cancer ward, acute hospital	Selected venous access devices should be locked with 0.9% saline when not in use	Cancer Nurses Society of Australia Vascular Access Guidelines (27)	TIVADs and tc-CICCs routinely locked with heparinised saline, in accordance with local policy	Health service policy changed to align with guidelines; policy updates circulated; staff education; physical changes to storage	% eligible devices locked with saline
9. Outpatient high-risk foot clinic	Timely access to high-risk foot clinic for patients with urgent referrals to podiatry	NADC (iHRFS) Standards Review Version 2.0 (28)	<20% of high-risk patients were receiving an appointment within 3 days	Schedule changes to preserve capacity and spread availability across the week; more phone reviews; more shared care with GPs	% high-risk patients seen <3 days
10. Surgical theatres tertiary hospital	Operating theatre temperatures at 23 °C during C-sections to prevent neonatal hypothermia	ERAS Society guideline for intraoperative care in caesarean delivery (29)	Average temperatures set at 19.5 °C; clinicians concerned about observed rates of neonatal hypothermia	Dissemination of evidence and lobbying/coordination of stakeholders (engineering, medical, management, and nursing leaders), new processes established	% X-sections with theatre temperature 21 °C–24°C

ANZMOSS, Australia and New Zealand Metabolic and Obesity Surgery Society; NADC (iHRFS), National Association of Diabetes Centres (Interdisciplinary Diabetes High-Risk Foot Services); ERAS, enhanced recovery after surgery; TIVAD, totally implantable venous access device; tc-CICC, tunnelled cuffed-centrally inserted central catheter; GP, general practitioner.

The projects fell into three categories based on the type of change required to align care with evidence. For five projects, evidence translation required a local policy change which, once implemented, could be followed as routine practice. Three projects required continuing behaviour change from clinicians, with the implementation of evidence into practice dependent on a choice to provide the evidence-based assessment or intervention at each patient encounter. Two are related to the provision of care within a given time frame, requiring changes to complex systems to enhance the flow of patients through services.

During the implementation phase of the projects, Project Leads used a range of different implementation strategies in their efforts to change practice. Most common strategies included staff training, development of resources, updates to local policy and processes, and recruitment of champions (Table 2). For example, project 4 provided staff training sessions on assessing cognition after stroke, compiled evidence-based resources on validated assessment tools, and used the existing staff supervision structure to reinforce learning. Project 8 focused on changing the hospital policy to align with evidence-based recommendations for locking of venous access devices, accompanied by staff education regarding the reason for the change and altering storage arrangements and signage for related supplies and equipment to prompt use of the correct venous locking procedure. Project Leads typically collected their pre-intervention data over a period of 4–8 weeks after workshop 2, and their post-intervention data 6–9 months later. Some flexibility was granted in the collection of post-intervention data to accommodate local work schedules and variation in the complexity of collecting outcome measures for the different projects.

### 3.3 Effectiveness

Significant changes in the odds of receiving evidence-based care were achieved by 8 of 10 projects. The outcomes for each of the 10 projects are summarised in Table 3.

**Table 3:** Outcome of research translation projects.

Project description	Pre-intervention			Post-intervention			Log odds ratio (95% CI)^a^
	*n*	Care aligned with clinical practice guideline		*n*	Care aligned with clinical practice guideline		
		Yes [*n* (%)]	No [*n* (%)]		Yes [*n* (%)]	No [*n* (%)]	
Use of visual menus in residential aged care	26	0 (0)	26 (100)	26	16 (62)	10 (39)	4.42 (1.52 to 7.33)^b^
Timing of sestamibi injection to parathyroid scintigraphy	51	9 (18)	42 (83)	45	44 (98)	1 (2)	5.32 (3.22 to 7.43)^b^
Post-bariatric surgery dietetics review <8 weeks	18	12 (67)	6 (33)	27	0 (0)	27 (100)	−4.66 (−7.61 to −1.71)
Cognitive assessment prior to hospital discharge after stroke	100	18 (18)	82 (82)	30	19 (63)	11 (27)	2.06 (1.16 to 2.96)^b^
Circuit training for patients during stroke rehabilitation	50	0 (0)	50 (100)	55	37 (67)	18 (33)	5.32 (2.48 to 8.16)^b^
Delivery of targeted education for falls prevention in hospital	98	25 (26)	73 (75)	10	8 (80)	2 (20)	2.46 (0.84 to 4.07)^b^
Gait speed and balance assessment in rehabilitation	50	4 (8)	46 (92)	50	30 (60)	20 (40)	2.85 (1.68 to 4.02)^b^
Recommended locking solution used for venous access devices	30	8 (27)	22 (73)	15	14 (93)	1 (7)	3.65 (1.47 to 5.83)^b^
Timely podiatry assessment for high-risk foot wounds	28	5 (18)	23 (82)	28	9 (32)	19 (68)	0.78 (−0.47 to 2.03)
Recommended theatre temperature for caesarean sections	9	1 (11)	8 (89)	22	15 (68)	7 (32)	2.84 (0.58 to 5.11)^b^
**Total**	**460**	**82 (18)**	**378 (82)**	**308**	**192 (62)**	**116 (38)**	**2.52 (0.93 to 4.11)**

a Log odds ratios, where values >0 indicate greater odds and values <0 indicate lesser odds of receiving evidence-based care post-intervention.

b Statistically significant increase in the odds of receiving evidence-based care post-intervention, at *p* < 0.05.

**Figure 2:**
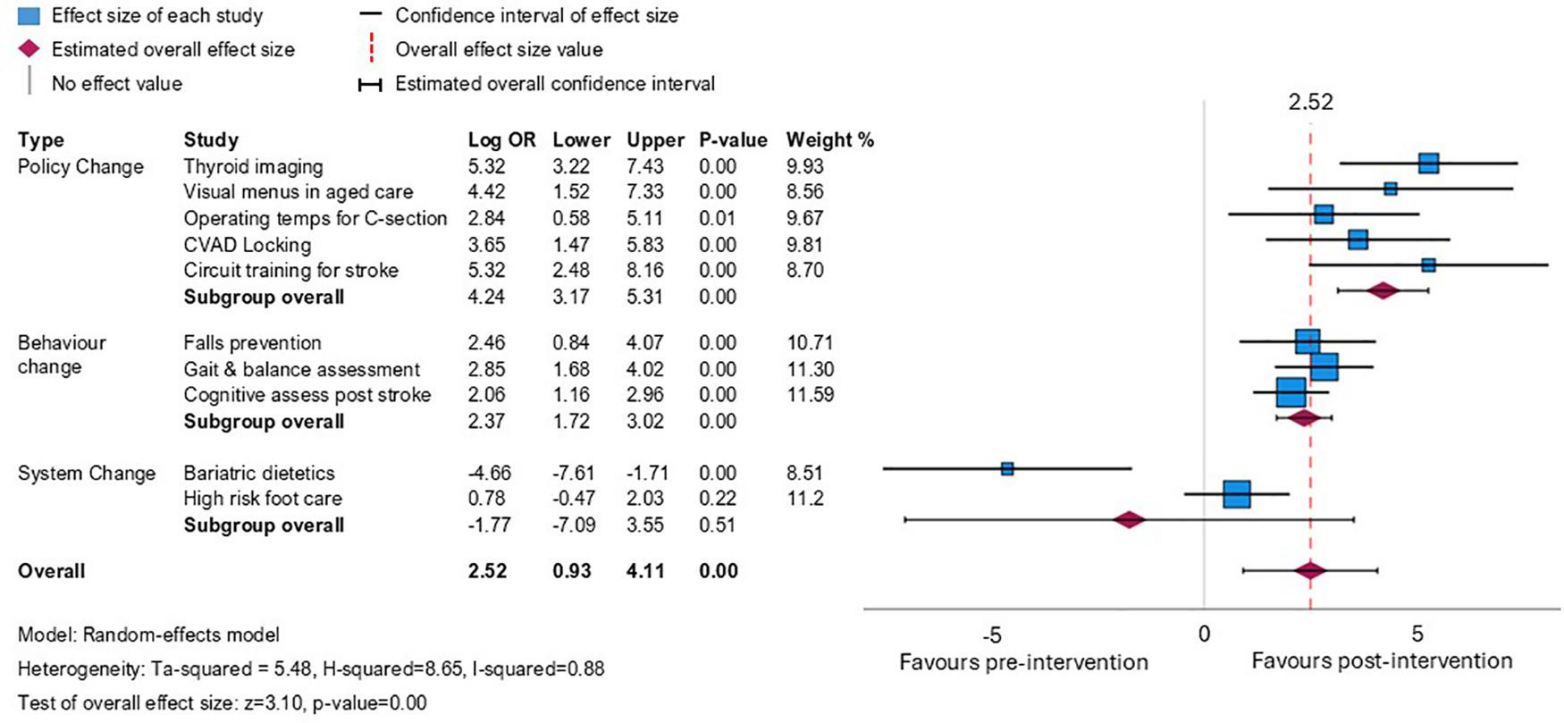
Meta-analysis showing odds ratio (log-transformed for symmetrical distribution around 0) for receipt of evidence-based care post- vs. pre-intervention across the 10 projects, presented by the type of intervention required to achieve a change in practice.

The planned pooled meta-analysis demonstrated that overall, the interventions appeared to have a strong positive effect (logOR 2.52, 95% CI: 0.93–4.11), but with high heterogeneity (*I*^2^ = 0.88) (Figure 2). This heterogeneity is explained when the projects are analysed by subgroup, based on the type of change required to align evidence with guidelines. Projects involving change targeted at the local policy level (*n* = 5 projects) resulted in patients having the highest odds of receiving evidence-based care after the completion of the evidence implementation projects compared with those seen before (logOR 4.65, 95% CI: 3.43–5.86, *I*^2^ = 0). Patients impacted by projects that required individual behaviour change (*n* = 3 projects) also had increased odds of care consistent with guidelines after project completion, but to a lesser degree (logOR 2.37, 95% CI: 1.72–3.02, *I*^2^ = 0). The two projects that aimed to improve the timing of clinical consultations to align with guidelines were less successful. One of these projects related to timely access to a high-risk foot clinic, where a small positive effect was observed but wide confidence intervals indicated uncertainty about this effect. In the other project, sustained increases in service demand during the trial period overwhelmed all strategies implemented to address the goal to improve timely care after bariatric surgery.

### 3.4 Level of evidence implementation

At baseline, six Project Leads described the level of implementation of the evidence in their area at a minimum of the equivalent of level 4 of Meyer's stages of healthcare implementation (describing the proposal having been formally proposed) with the highest degree of implementation reported as level 6 (describing evaluation undertaken at a political or strategic level). At follow-up, Project Leads reported that the level of implementation of evidence was at either the trial (level 7) or acceptance (level 8) level (Supplementary Figure S3).

### 3.5 Confidence in research translation

Self-reported responses on the Confidence in Research Translation Questionnaire were completed both at baseline and follow-up by at least one Project Lead from each completed project (*n* = 11 Project Leads, Table 4). Participants demonstrated significant increases in confidence in five out of eight domains of research confidence. Areas in which scores did not increase included confidence in adapting knowledge, which was already high at baseline (*p* = 0.58); confidence in sustaining change, which may have been impacted by the limited time frame of the training programme (*p* = 0.22); and confidence in identifying, reviewing, and selecting knowledge (*p* = 0.06).

**Table 4:** Changes in confidence in research translation before and after the programme.

Confidence in research translation questionnaire subscale	Pre-median (IQR) (*n* = 13)	Post-median (IQR) (*n* = 11)	Wilcoxon signed-rank test (*z*-score, *p*-value)
Identifying problems	3.5 (3.25–4.0)	5.0 (4.0–5.0)	2.46, *p* = 0.01
2. Identify, review, and select knowledge	3.2 (2.4–3.75)	3.7 (3.3–3.8)	1.91, *p* = 0.06
3. Adapting knowledge	4.0 (3.5–5.0)	4.0 (4.0–4.8)	0.55, *p* = 0.58
4. Assessing barriers	3.0 (3.0–3.5)	4.5 (4.0–5.0)	2.82, *p* < 0.01
5. Implementing interventions	3.4 (2.6–3.8)	4.0 (3.8–4.8)	2.68, *p* < 0.01
6. Monitoring change	3.0 (2.7–4.0)	4.0 (3.7–5.0)	2.68, *p* < 0.01
7. Evaluation of change	2.7 (2.3–3.0)	3.7 (3.3–4.0)	2.45, *p* = 0.01
8. Sustaining change	3.7 (3.3–4.0)	4.0 (3.7–4.0)	1.23, *p* = 0.21

Responses provided to 26 questions across eight subscales on a Likert scale in response to the question ‘I am confident that I can…’ followed by response options ‘strongly agree’, ‘agree’, ‘neither agree nor disagree’, ‘disagree’, and ‘strongly disagree’. Higher scores indicate higher levels of confidence. Multiple questions contribute to each of the eight subscales shown, with subscale scores calculated from the mean of the response scores for the contributing questions (2).

Wilcoxon signed-rank test based on *n* = 11 pairs with complete data.

## 4 Discussion

In this study, we tested the proof of concept of a deliberate, novel strategy to implement evidence into practice using a training and support programme for motivated healthcare professionals, structured around an adaptation of A3 Lean Improvement methodology for evidence implementation projects. Results from 10 pilot projects evaluated on over 700 patients using the ART-A3 indicate that clinician-led projects, supported by a structured approach to implementation methods, small group training, and mentoring, can lead to significant increases in the proportion of patients receiving care aligned with clinical practice guidelines. These methods may be better suited to some types of change processes than others, but all clinician Project Leads reported increased confidence in research translation after participation in the programme.

Yawning chasms between evidence and practice in healthcare are well-known (30), and much has been learned about how to address the problem through the emergence of implementation science (31). However, literature to date has been dominated by papers describing theoretical approaches to addressing implementation challenges (9, 14) and studies describing the outcomes of implementation initiatives targeting specific problems, services, or programmes (32). For evidence practice gaps to be addressed effectively, rapidly, continuously, and at scale, we need practical methods to integrate this work into the day-to-day business of healthcare design and delivery. Furthermore, solutions need to be low-cost and adaptable across different health systems to enable effective evidence translation in high- and low-resource healthcare systems alike. This study addresses that gap by describing an approach based on the familiar A3 problem-solving approach for continuous quality improvement that can be integrated into existing systems. This tool can then be used as a scaffold for introducing other theories and frameworks [such as the COM-B behaviour change model (15) or AACTT approach to selecting implementation interventions (16)] specific to implementation science that can be used selectively to help identify barriers, choose interventions, or evaluate outcomes. This approach harnesses time already devoted to continuous quality improvement to target evidence–practice gaps in a systematic and ongoing manner.

While the current study was designed as a proof-of-concept trial, results provide preliminary evidence that the ART programme increased the proportion of patients receiving care in accordance with clinical practice guidelines in the clinical areas of a large health network in which the projects were conducted. The 10 test projects were diverse, suggesting the method is suitable for application across the breadth of very large health networks and clinical specialties. However, the methods as currently presented may be better suited to some types of evidence gaps than others. The most successful projects were those in which the closing of the evidence–practice gap required a local change to policy and procedure: for example, a new policy for locking of venous access devices, with associated changes to storage of stock and instructions to staff, or a change to workflow to enable correct timing of test procedures in parathyroid imaging (33). For each of these projects, effort was required to initiate the change, develop and approve new policies, educate staff, and ensure the required resources, equipment, and infrastructure were in place to support the new process. However, once established, new patients entering the service were exposed to an environment in which evidence-based care was built into the service design.

Evidence–practice gaps requiring behaviour change from individual staff were also successful, albeit with smaller effect sizes. In these cases, Project Leads were implementing strategies to encourage clinicians to choose to offer assessments or treatments aligned with guidelines: balance and gait assessment or tailored education to prevent falls or cognitive screening assessments for all stroke survivors prior to hospital discharge. Behaviour change in healthcare is challenging, and previous researchers have also described the outcomes of interventions targeting the behaviour of health professionals to be meaningful but modest (34, 35). A systematic review evaluating interventions that focus on changing the behaviour of service providers noted that 26 of 28 studies that described contextually relevant interventions tailored to the population were successful in changing behaviour and that interventions that were education-based were associated with better outcomes if they were interactive, had credible instructors, and offered extended follow-up (32). This suggests that there may be opportunities for improvement of the first iteration of the ART programme tested in this trial, through supporting participants to identify the level of behaviour change required to implement the guideline recommendation of interest. Rather than concluding that the ART strategy is less successful for evidence gaps requiring clinician behaviour change, it is likely that this issue could be addressed with a greater focus on evidence-based behaviour change strategies within the training programme.

The two projects in our sample did not result in significant improvements in evidence-based care related to timely access to treatment. In both cases, the evidence–practice gap identified is related to the time between a health event (disease identification or surgery) and specialist follow-up by an allied health professional (podiatrist or dietitian). The changes required to achieve the desired outcomes involved complex systems, with many factors outside the control of the Project Leads. The framework for developing and evaluating complex interventions published by the United Kingdom Medical Research Council describes a core set of elements for consideration, including context, theory, stakeholders, uncertainties, and economic considerations (36). The elements interact with multiple other domains related to feasibility, implementation, and evaluation. While the Project Leads in both cases implemented changes to enhance the service and increased their confidence in research translation, the resources provided through the ART programme may not have been sufficient to address gaps of this nature within the available timeframe.

A key component of the ART strategy was the adaptation of the existing A3 problem-solving tool that was already in use at the health organisation where the study was conducted. We hypothesise that this contributed to the success of the strategy in three ways. Firstly, the original document was familiar to the clinicians in the study, providing immediate comfort and confidence that they were embarking on something achievable (12). Secondly, the A3 was already accepted and readily understood by the health organisation leadership, enabling the ART-A3 and accompanying supports to be easily integrated into existing systems. Finally, in concordance with an issue identified by previous researchers (32), the ART-A3 concept provided a simple way to conceptualise the steps required to translate evidence into practice, while enabling the demystification of implementation science. Project Leads were able to logically work through the steps to appraise the evidence, set a target, understand barriers to research translation, and design and implement strategies consistent with theoretical frameworks informed by implementation science literature.

This trial was intended as a proof-of-concept study and has some limitations. Project Leads collected their pre- and post-implementation data, and it is possible that the lack of blinding of those collecting the data created a source of bias or led to a Hawthorne effect. While this was partially mitigated by the nature of the data (objective audits rather than subjective outcome measures), it is a limitation that should be addressed in future studies. The use of historical control data in the pre-post design did not account for seasonal factors or other health service factors that could affect outcomes. Future larger studies should incorporate designs that would take into account these factors. Results within each trial did not consider gender differences, but the diversity of the test projects suggests that these methods can be used to tackle evidence-translation gaps across a broad range of patient populations. The sample size of 10 projects and 13 participants and relatively small audit samples for some projects could be considered a further limitation, although the sample of 10 projects was above recommendations for a planned pooled analysis of this nature. Each Project Lead calculated the sample size required to detect change based on expected proportions of patients receiving evidence-based care relevant to their population and setting, resulting in variable sample sizes across the projects, with associated influence on the outcomes of the meta-analysis. While it is reasonable to determine sample size based on individual projects based on the research question and size of the desired effect, consistent audit sample sizes across projects would have strengthened the meta-analysis and could be a consideration for future studies.

Our decision to combine the results of the studies in a planned pooled meta-analysis could also be questioned, given the diversity of the projects. However, combining 10 project outcomes in a single analysis dilutes the potential influence of bias within any individual project, and subgroup analysis according to the type of intervention confirmed very low levels of statistical heterogeneity. Positive outcomes across a diverse range of projects also suggest promise for external validity, despite this proof-of-concept study being conducted in one health service in Australia. Furthermore, the results are intended to illustrate the effect sizes across the projects, and a high degree of statistical homogeneity in the findings within projects that involved similar types of change processes supports the appropriateness of this approach. Many of these limitations are those that might be expected from a proof-of-concept study designed to provide preliminary evidence about a novel way to translate evidence into practice in health services. A fully powered trial with robust design incorporating both effectiveness and implementation outcomes and sufficient follow-up time to evaluate sustainability is needed to determine potential for implementation at scale.

## 5 Conclusion

As new evidence emerges in healthcare, systematic methods to address evidence-based practice gaps are vital. Isolated projects dependent on the involvement of experienced implementation scientists will not be enough—the implementation of evidence into practice needs to be integrated into the routine work of service providers. While it has some limitations, this small proof-of-concept study suggests that the concept of the ART-A3, accompanied by training and support systems, may be a promising mechanism to demystify implementation science and address local evidence practice gaps at an industrial scale.

## Data Availability

The raw data supporting the conclusions of this article will be made available by the authors, without undue reservation.
