# Supplementary material for: The ART of evidence-based care: proof of concept of a novel strategy to accelerate research translation in health organisations
Source: Front Health Serv. 2026 Jan 12;5:1691307. doi: 10.3389/frhs.2025.1691307 (PMC12832972; doi:10.3389/frhs.2025.1691307)
Supplement: Supplementary file 1 [file Table1.docx]

Supplementary Material

# Contents

**Tables**

Supplementary Table 1: Workshop outline p. 2

Supplementary Table 2: Intervention described using TiDIER checklist p. 3

Supplementary Table 3: Confidence in Research translation questionnaire p. 4

**Figures**

Supplementary Figure 1: ART-A3 template with guidance for clinicians p. 5

Supplementary Figure 2: Stages of Healthcare Implementation Scale p. 6

Supplementary Figure 3: Level of implementation recorded at baseline and p. 7
after completion of the ART projects

Supplementary Table 1: Workshop outline

| **Workshop** | **ART-A3 components** | **Topics Covered** |
| --- | --- | --- |
| 1 (week 1) | Steps 1  and 2, and planning  for 7 | - Introduction of the ART-A3 and supporting resources - Evidence-practice gaps and levels of evidence - Define the target population and scope of the project - Define outcome measures and the goal/target - Governance and ethical review requirements for different types of project work |
| 2 (week 3) | Steps 3  and 4 | - Key concepts in implementation science - Ways to explore barriers and facilitators to implementation of evidence into practice - Plan “current state evaluation” and collection of baseline data |
| 3 (week 8) | Steps 5  and 6 | - Methods to review and synthesise data from current state evaluation - Strategies for implementation of evidence into practice - Development of an evidence implementation translation strategy consistent with the selected CPG |
| 4 (week 30) | Steps 7  and 8 | - Sharing of data and experiences of project implementation - Simple methods for analysis of data using free online statistics tools - Dissemination planning |

Supplementary Table 2: Description of the Accelerating Research Translation intervention using the TIDieR template

| Item | Description |
| --- | --- |
| Brief name | ART Framework |
| Why | Adapting tools already familiar to clinicians provides an opportunity to provide accessible, evidence-based guidance in the conduct of projects that aim to address evidence-practice gaps in health services. |
| What  (Materials) | ART-A3: An adaptation of the A3 project template often used for quality improvement in health services  Written resources (reading materials, slide deck, practical exercises) |
| What  (Procedures) | Workshops covering project planning, implementation science theory and evaluation.  Access to a project mentor  Implementation of a project addressing an evidence-practice gap |
| Who  provided | Workshop leader: Experienced researcher and implementation scientist with clinical background as an occupational therapist, who holds a conjoint academic appointment between the health service and university partner  Mentors: Academic or clinician researchers with experience in implementation science, from allied health, nursing or medical professions. |
| How | Combination of face to face and online modes of delivery. |
| Where | Face to face workshops conducted in central locations within the health service, with remote participation accommodated with videoconferencing.  Mentor meetings conducted at mutually agreed locations or online.  Projects conducted in the participants local work area. |
| When and  How Much | 4 x 2.5 hour workshops (3 over the first 2 months, the fourth after 6 months)  Mentor meetings suggested monthly, scheduled by mutual agreement. |
| Tailoring | The ART-A3 was adapted from the traditional A3 used for quality improvement projects within this health service.  Personalised support was provided to each Project Lead by the mentor.  All Project Leads received the same workshop training. |
| Modifications | Due to variations in the timelines required by participants to finish their projects, some flexibility was offered in the timing for collection of follow up data. Follow up data for all projects was collected between 8 and 12 months following the commencement of the training. |
| How well  (planned) | Attendance at workshops was tracked.  Participants provided copies of their completed ART-A3 project reports summarising progress at each stage of the project. |
| How well  (actual) | All participants attended a minimum of 3 of the 4 workshops, and all made arrangements to view recorded material for any workshops missed.  Completed ART-A3 project reports were submitted for all completed projects. |

Supplementary Table 3: Confidence in Research translation questionnaire

|  | **I am confident that I can…** | Strongly disagree | Disagree | Neither agree/ disagree | Agree | Strongly agree |
| --- | --- | --- | --- | --- | --- | --- |
| **Identify problem** | Identify and generate a clinical question |  |  |  |  |  |
|  | Collect patient data based on clinical question |  |  |  |  |  |
| **Identify, review, select knowledge** | Formulate a question to guide a literature review |  |  |  |  |  |
|  | Conduct a literature review |  |  |  |  |  |
|  | Search best evidence to answer a clinical question |  |  |  |  |  |
|  | Critically appraise the evidence |  |  |  |  |  |
|  | Interpret study results obtained by statistical tests |  |  |  |  | **`** |
|  | Determine the clinical significance of study tests |  |  |  |  |  |
| **Adapt knowledge** | Determine if evidence applies to my patients/contexts |  |  |  |  |  |
|  | Use evidence to decide on an appropriate course of action |  |  |  |  |  |
| **Assess Barriers** | Assess local context for barriers and enablers |  |  |  |  |  |
|  | Use research translation frameworks / theories to assess contexts |  |  |  |  |  |
| **Select, implement interventions** | Implement knowledge where I work |  |  |  |  |  |
|  | Overcome local barriers to implement knowledge |  |  |  |  |  |
|  | Select and apply a range of implementation strategies |  |  |  |  |  |
|  | Implement a research translation project in a timely and efficient way |  |  |  |  |  |
|  | Access the best resources to implement a research translation project |  |  |  |  |  |
| **Monitor** | Develop a plan to monitor implementation |  |  |  |  |  |
|  | Measure and collect data on a patient problem |  |  |  |  |  |
|  | Use patient data to review research translation activities and progress |  |  |  |  |  |
| **Evaluate** | Select and measure appropriate evaluation outcome |  |  |  |  |  |
|  | Analyse quantitative research data |  |  |  |  |  |
|  | Analyse qualitative research data |  |  |  |  |  |
| **Sustain, disseminate** | Share the data I collect with colleagues and end users |  |  |  |  |  |
|  | Write for publication in peer-reviewed journals |  |  |  |  |  |
|  | Promote ongoing use of research translation to colleagues |  |  |  |  |  |

Adapted from Young AMP, Olenski SP, et al. Knowledge Translation in Dietetics: A Survey of Dietitians' Awareness and Confidence. *Canadian Journal of Dietetic Practice and Research.* 2020;81(1):49-53. Terminology has been changed from “knowledge translation” to “research translation” for consistency.


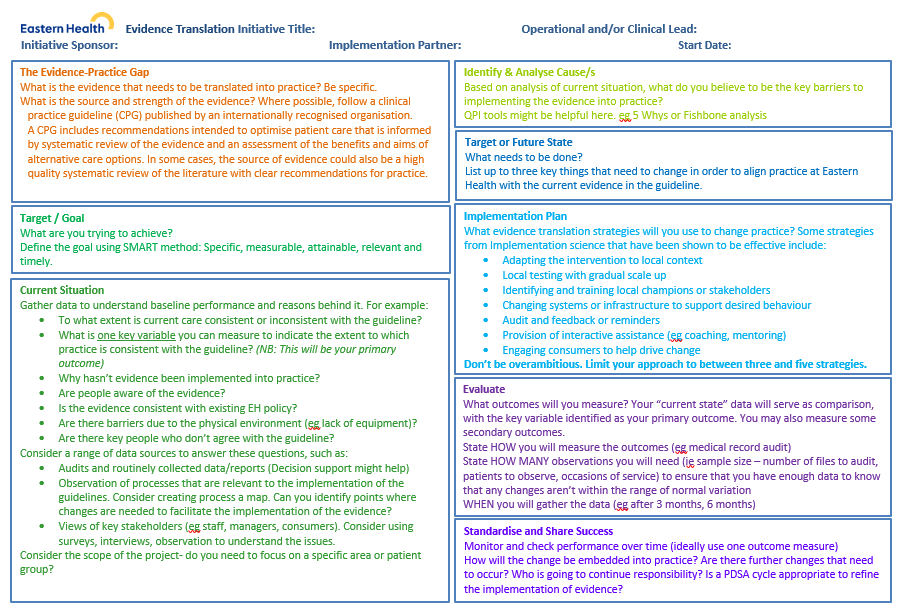
Supplementary Figure 1: ART-A3 template with guidance for Project Leads

Supplementary Figure 2: Stages of Healthcare Implementation described by Myer and Goes (1988)^*^ describing the processes that services use to adopt new technologies
(original wording in bold, word substitutions in italics)

**Knowledge-Awareness Stage**

1. Apprehension. Individual organization members learn of an innovation’s existence *(Individual organization members learn of an evidence practice gap).*

2. Consideration. Individuals consider the innovation suitable for their organization.
*(Individuals have considered whether this evidence-based guideline is suitable for the work area)*

3. Discussion. Individuals engage in conversations concerning adoption.
*(Individuals have engaged in conversations concerning adoption of the evidence-based guideline)*

**Evaluation-Choice Stage**

4. Acquisition proposal. Adoption of the innovation is proposed formally.
*(Adoption of the evidence-based guideline has been proposed formally)*

5. Medical-fiscal evaluation. The proposed innovation is evaluated according to medical and financial criteria.
*(The proposed evidence-based guideline has been evaluated according to medical and financial criteria)*

6. Political-strategic evaluation. The proposed innovation is evaluated according to political and strategic criteria.
*(The proposed evidence-based guideline has been evaluated according to policatical and strategic criteria*

**Adoption-Implementation Stage**

7. Trial. The innovation *is implemented* but is still under trial evaluation.
*(The proposed evidence-based guideline has been implemented but is still under trial evaluation)*

8. Acceptance. The innovation becomes well accepted and frequently used.
*(The proposed evidence-based guideline becomes well accepted and frequently used)*

9. Expansion. The innovation is expanded, upgraded, or replaced with a *based on updated evidence*.
*(The proposed evidence-based guideline has been expanded, upgraded, or replaced with a based on updated evidence)*

* Meyer AD, Goes JB. Organizational Assimilation of Innovations: A Multilevel Contextual Analysis. Academy of Management Journal. 1988;31(4):897-923


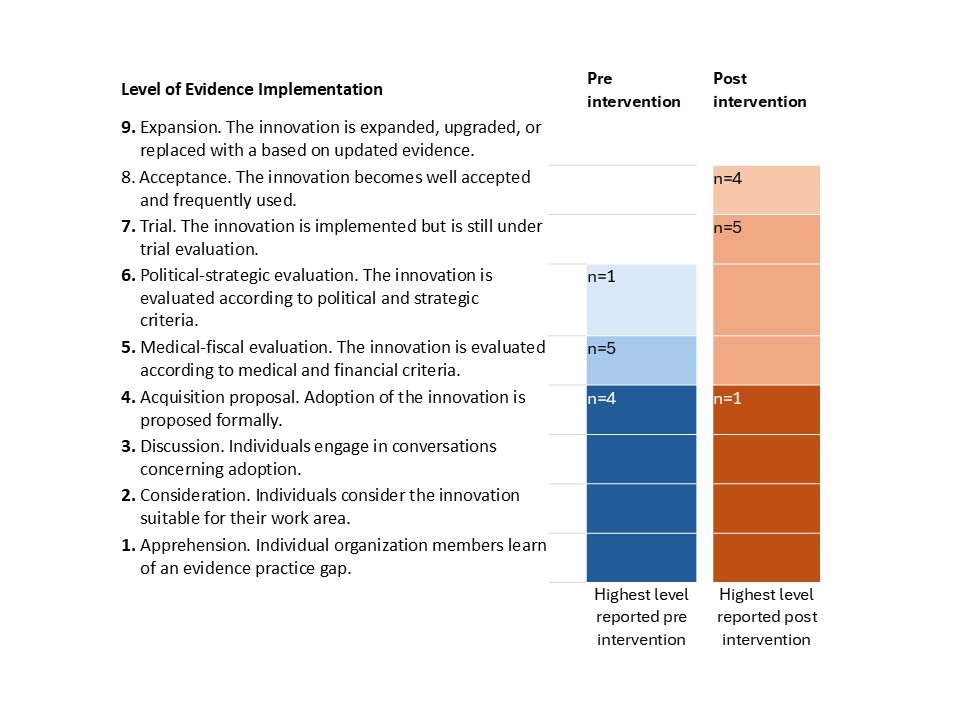
Supplementary Figure 3: Change in level of implementation of evidence before and after intervention, measured against the Stages of Healthcare Implementation (Meyer and Goes, 1988)
